# Machine Learning‐Guided Identification of Madecassoside as a Potential RAD51 Modulator That Sensitizes Breast Cancer Cells to Cisplatin

**DOI:** 10.1111/cbdd.70416

**Published:** 2026-10-13

**Authors:** Minju Oh, Mi‐Young Jeong, Jiwon Choi

**Affiliations:** ^1^ College of Pharmacy Dongduk Women's University Seoul South Korea

## Abstract

Homologous recombination (HR) is an essential pathway for preserving the genomic stability of cells by repairing DNA damage, especially double‐strand breaks and interstrand crosslinks. RAD51 recombinase plays a central role in the HR pathway, and when DNA damage occurs, RAD51 binds to single‐stranded DNA at the cleavage site to form a helical nucleoprotein filament. Therefore, pharmacological inhibition of RAD51 is emerging as an attractive strategy to block the repair mechanisms of cancer cells and maximize the therapeutic efficacy of conventional radiation and chemotherapy. Several RAD51 small‐molecule inhibitors, including CYT‐0851, B02, and IBR2, have demonstrated preclinical or early clinical efficacy, but no candidate has yet received regulatory approval. In this study, we identified a novel RAD51 inhibitor from a natural product library using an in silico workflow that combines machine learning models and molecular docking simulations. Among the five machine learning models evaluated, the random forest (RF) model showed the best overall predictive performance after SMOTE‐based class balancing and near‐zero variance filtering, achieving an accuracy of 0.933, sensitivity of 0.800, specificity of 1.000, Cohen's kappa of 0.842, and ROC‐AUC of 0.980. Using an optimized RF model, 1142 natural compounds were virtually screened, and Madecassoside was finally selected by molecular docking analysis. Biological validation showed that MDA‐MB‐231 cells increased susceptibility to cisplatin after Madecassoside treatment and also markedly inhibited the formation of RAD51 foci induced by cisplatin, strongly inhibiting DNA damage repair. These findings suggest that Madecassoside is a promising RAD51‐modulating lead compound that may enhance cisplatin sensitivity by impairing RAD51‐mediated DNA repair.

## Graphical abstract

Madecassoside was selected from an in‐house natural product library through machine learning‐based virtual screening and identified as a potential modulator of RAD51‐mediated DNA repair that enhances cisplatin sensitivity in breast cancer cells.

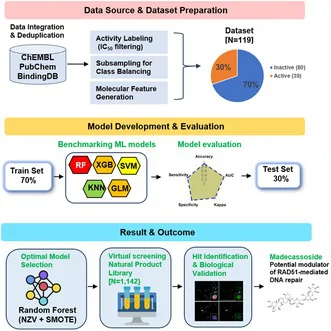

## 1 Introduction

The cellular genome is constantly damaged by various exogenous and endogenous factors, including DNA double‐strand breaks, which can cause cell death or cancer‐causing mutations. Cells repair DNA using non‐homologous end joining (NHEJ) and homologous recombination (HR) pathways (Shkundina et al. 2021; Mehta and Haber 2014; Bonilla et al. 2020; Gupta et al. 1997). HR uses sister chromatids as templates to restore DNA to its original sequence and maintain genomic integrity (Shkundina et al. 2021; Sung 1994; Demeyer et al. 2021). RAD51 recombinase is a key protein in genetically conserved eukaryotic cells and plays a central role in the HR pathway. When DNA damage occurs, RAD51 binds to single‐stranded DNA (ssDNA) at the cleavage site to form a helical nucleoprotein filament (Bonilla et al. 2020; McIlwraith et al. 2000). The resulting filaments explore homologous DNA sequences and induce strand invasion to form a D‐loop structure that initiates full‐scale DNA synthesis and repair. Numerous studies have reported abnormal overexpression of RAD51 in a variety of malignant tumors, including breast, ovarian, liver, and pancreatic cancers (Lynch et al. 2021; Wang et al. 2022; Gu et al. 2022; Budke et al. 2016). Cancer cells use RAD51 to withstand high levels of replication stress and to resist and survive treatment by quickly repairing radiation‐ or chemotherapy‐induced DNA damage. Therefore, RAD51 has attracted attention as a promising therapeutic target for blocking the survival of cancer cells and maximizing the efficacy of conventional treatments. In addition, as RAD51 is involved in viral replication and propagation mechanisms, its application is expanding beyond anticancer drugs to a wide range of antiviral drugs (Pham et al. 2023; Pasaje et al. 2011; Cosnefroy et al. 2012). To date, small‐molecule inhibitors such as B02, RI‐1, and IBR2 have been developed to inhibit the activity of RAD51 (Pan et al. 2025; Richardson 2005; Hong et al. 2023; Budke et al. 2012). They mainly block HR by inhibiting the ssDNA‐binding force of RAD51 or by interfering with protein‐to‐protein multimerization. However, many of these compounds have limitations in that they do not achieve sufficient pharmacokinetic properties or selectivity to proceed to the clinical stage. To overcome this limitation, this study aimed to identify RAD51 target compounds with structural diversity and robust efficacy by integrating machine learning strategies and structure‐based design. Recent advances in machine learning and artificial intelligence have accelerated data‐driven prediction and decision‐making across diverse areas of chemical, biological, and environmental research. In particular, studies predicting changes in cancer cell migration after natural product treatment (Umar, Rizaner, et al. 2023; Umar, Aliyu, et al. 2023), as well as AI‐based analyses of complex resource‐recovery systems, demonstrate the broad applicability of these approaches (Edo et al. 2026). Collectively, these studies support the use of machine learning as a useful tool for identifying biologically relevant compounds and selecting candidates for experimental validation. In this study, five machine learning algorithms (RF, XGBoost, SVM, KNN, and GLM) were trained and evaluated using data from ChEMBL, PubChem, and BindingDB. The optimized model was then applied to an in‐house natural product library, and selected candidates were further evaluated by molecular docking and biological validation. Through this workflow, Madecassoside was identified as a promising RAD51‐modulating candidate that enhanced cisplatin susceptibility and suppressed cisplatin‐induced RAD51 foci formation in MDA‐MB‐231 breast cancer cells. The overall strategy employed in this study, encompassing data processing, model development, and validation, is summarized in the workflow presented in Figure 1. These results highlighted that the combination of machine learning and structure‐based drug discovery is an effective strategy for discovering novel RAD51‐targeting inhibitors.

**FIGURE 1:**
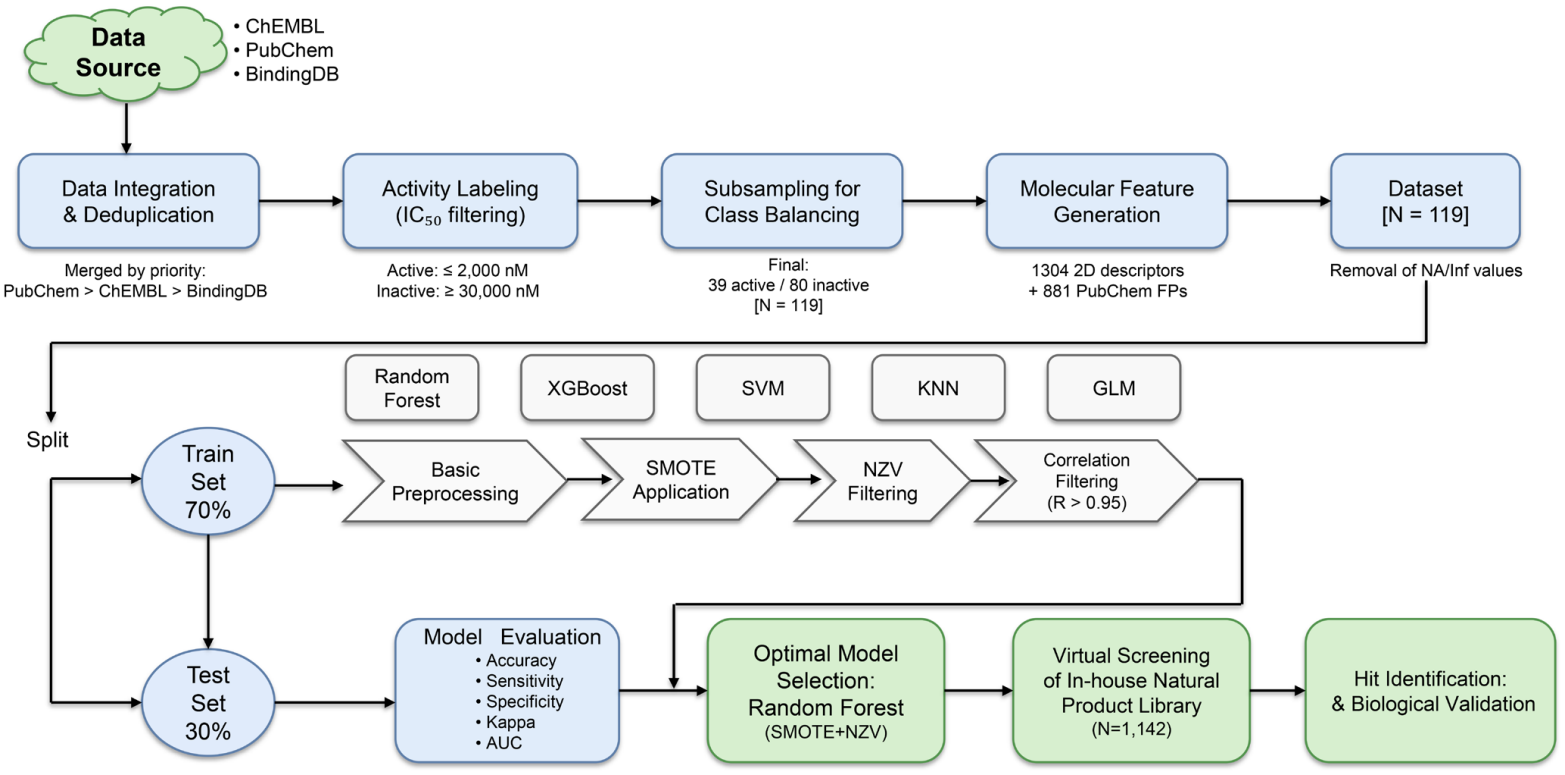
Systematic workflow for the identification of novel RAD51 inhibitors. This integrated pipeline consists of four main steps: (1) data curation, including data integration, deduplication, activity labeling, and class balancing, using RAD51 inhibitor data from ChEMBL, PubChem, and BindingDB (*N* = 119); (2) feature engineering using 1304 2D descriptors and 881 PubChem fingerprints; (3) model optimization by comparing five machine learning algorithms while sequentially applying SMOTE‐based class balancing, near‐zero variance filtering, and correlation‐based feature elimination; and (4) virtual screening of an in‐house natural product library using the optimized Random Forest model followed by experimental biological validation.

## 2 Materials and Methods

### 2.1 Data Collection and Curation

Bioactivity data for DNA repair protein RAD51 homolog 1 (UniProt ID: Q06609; ChEMBL ID: 2034807) was obtained from three public data sources: PubChem, ChEMBL, and BindingDB (Zdrazil et al. 2024). Only records with reliable IC_50_ values and structural information were included in the final dataset. Redundant entries across the three public databases were identified using canonical SMILES and were removed according to the database priority order: PubChem (priority 1), ChEMBL (priority 2), and BindingDB (priority 3). The final curated dataset consisted of 626 unique compounds. The results of the filtering and curation processes are detailed in Table 1. The activity threshold was defined as IC_50_ ≤ 2000 nM for the active class and IC_50_ ≥ 30,000 nM for the inactive class. Finally, molecules with IC_50_ values between 2000 and 30,000 nM were removed to ensure a clear separation for model training. To optimize predictive performance, a preprocessing pipeline was applied before model training, including SMOTE (Synthetic Minority Over‐sampling Technique)‐based class balancing, near‐zero variance (NZV) filtering, and correlation filtering. This preprocessing strategy helped reduce class imbalance and feature redundancy, allowing the models to learn from more informative variables.

**TABLE 1:** Summary of RAD51 bioactivity data collected and filtered from PubChem, ChEMBL, and BindingDB.

Database source	Number of compounds	Filtered unique compounds	Data retrieval date
PubChem	199,399	312	December 31, 2024
ChEMBL	290	136	December 24, 2024
BindingDB	541	541	February 13, 2024

### 2.2 Model Development and Training Strategy

To establish and validate the predictive model, we divided the curated dataset into a training set (70%) and a test set (30%). Molecular descriptors and fingerprints were calculated using PaDEL‐Descriptor software (Yap 2011) with the PowerShell command‐line interface in a Python environment. For molecular representation, 1304 two‐dimensional (2D) descriptors and 881 PubChem fingerprints were calculated to construct 2185 molecular properties. To ensure reproducibility, all modeling procedures were performed using a fixed random seed of 1234. To explore the optimal classification model for predicting RAD51 inhibitory activity, we compared and evaluated the performance of five machine learning algorithms: Random Forest (RF; Breiman 2001), XGBoost (Chen and Guestrin 2016), Support Vector Machine (SVM) (Cortes and Vapnik 1995), k‐nearest neighbor (KNN) (Cover and Hart 1967), and Generalized Linear Model (GLM) (Nelder and Wedderburn 1972). The performance of each machine learning model was evaluated across four cumulative preprocessing pipelines: Basic, Basic + SMOTE, Basic + SMOTE + near‐zero variance (NZV) filtering, and Basic + SMOTE + NZV + correlation‐based feature removal using a threshold of *r* ≥ 0.95. All analyses were performed using R (version 4.4.1) to train and evaluate the model performance using the Caret package (version 7.0‐1) (Kuhn 2008). The algorithm was run using each of the following R libraries: RF (version 4.7‐1.2) for RF, xgboost (version 1.7.8.1) for XGBoost, e1071 (version 1.7‐16) for SVM, caret (version 6.0‐96) for KNN, and the base stats package for GLM.

### 2.3 Model Evaluation and Performance Metrics

The classification performance of the model was evaluated using several standard metrics derived from confusion matrices, including Accuracy, Sensitivity, Specificity, Cohen's Kappa (*κ*), and Area under the ROC curve (ROC‐AUC) (Cohen 1960). Accuracy was defined as the proportion of correctly classified instances among all test samples. Sensitivity and specificity were used to evaluate the ability of the model to accurately identify active and inactive compounds, respectively (Fawcett 2006). In particular, considering the class imbalance characteristics of datasets, Cohen Kappa coefficients (*κ*) were calculated to strictly rule out the possibility that predictions and real classifications match by chance. The ROC‐AUC was used to evaluate the overall discrimination power of each model across different classification thresholds. Model performance was first estimated by fivefold cross‐validation of the training dataset, and the final performance metric was derived using an independent test dataset based on a classification probability threshold of 0.5. All statistical analyses and modeling were performed using the cart (version 6.0‐96) and pROC packages in the R software environment.

### 2.4 Virtual Screening and External Validation

To identify novel RAD51 inhibitors, virtual screening was performed using an in‐house natural product library of approximately 2320 compounds. After salts and duplicate records were removed, 1142 unique natural products were retained based on canonical SMILES. For each compound, 1304 two‐dimensional (2D) molecular descriptors and 881 PubChem fingerprints were computed using PaDEL‐Descriptor software (version 2.21). As with the training dataset, missing values were replaced by 0, and non‐finite values (Inf, ‐Inf, NA) were deleted. To ensure comparability, the same standardization protocol was applied to the training dataset and all virtual screening libraries were used for prospective compound prioritization. The predictive performance was evaluated using an optimized RF model. Biological activity predictions were generated using class probabilities (type = “probe”). Compounds with a predicted probability ≥ 0.5 were classified as active. Subsequently, the top ten candidates with high probability scores were selected for further molecular docking validation.

### 2.5 Molecular Docking Validation

Molecular docking simulations were performed using AutoDock Vina (version 1.1.2) to evaluate the binding interactions between the two selected compounds (Geraniin and Madecassoside) and RAD51 (Kuhn 2008). The cryo‐EM structure of human RAD51 on ssDNA (PDB ID: 5NP7) was obtained from the RCSB Protein Data Bank and was used as a receptor model. Protein preparation was performed by removing crystallographic water molecules, adding polar hydrogen atoms, and assigning Kollman charges using AutoDock Tools. The 10 predicted compounds were converted from 2D to 3D ligand structures and subjected to energy minimization. The grid parameters for the ANP binding site on the Rad 51 protein include *x* = 9.188 Å, *y* = 7.204 Å, and *z* = 29.560 Å. We performed docking experiments using the active site of the ANP‐binding site. For subsequent analyses, the two compounds with the lowest binding affinities (kcal/mol) (Geraniin and Madecassoside) were selected. The selected binding poses were analyzed using PLIP (Protein‐Ligand Interaction Profiler) to characterize non‐covalent interactions and identify key binding residues (Salentin et al. 2015).

### 2.6 Cell Viability Assay

MDA‐MB‐231 cells were obtained from the Korean Cell Line Bank (Seoul, Republic of Korea). Cells were cultured in complete Roswell Park Memorial Institute (RPMI) 1640 medium (HyClone) supplemented with 10% fetal bovine serum (HyClone) and 1% penicillin–streptomycin (10,000 U/mL; Gibco) in a humidified incubator at 37°C with 5% CO_2_. Madecassoside was purchased from Sigma‐Aldrich. Cisplatin was obtained from Sigma‐Aldrich and reconstituted in phosphate‐buffered saline (PBS). In experiments with cells, Madecassoside was dissolved in DMSO. MDA‐MB‐231 cells were seeded into 96‐well plates at a density of 5 × 10^3^ cells per well and treated with varying concentrations of Madecassoside for 48 h. Cell viability was assessed using the Cell Counting Kit‐8 (CCK‐8; #CK04‐11, Dojindo) according to the manufacturer's instructions, and optical density (OD) was measured at 450 nM. Cells treated with complete medium containing 0.1% dimethyl sulfoxide (DMSO) served as the control group. Cell viability was expressed as a percentage of the control.

### 2.7 Clonogenic Survival Assay

Clonogenic survival assays were performed as described previously to assess the sensitivity of MDA‐MB‐231 cells to DNA‐damaging agents in the presence or absence of Madecassoside (Huang et al. 2012). Briefly, MDA‐MB‐231 cells were seeded at a density of 5 × 10^2^ cells/well in 6‐well plates. After overnight culture, cells were pretreated with Madecassoside (5 μM) in complete RPMI medium for 1 h. Cisplatin was diluted in PBS and added to cells at the indicated final concentrations. Following 1 h exposure, cells were washed three times with PBS and incubated in fresh medium containing Madecassoside (5 μM). After 7 days, the colonies were fixed and stained with a solution containing 0.05% crystal violet and 50% methanol in PBS. The number of colonies was quantified using ImageJ software.

### 2.8 Immunofluorescence Staining

To analyze RAD51 foci formation, MDA‐MB‐231 cells in the logarithmic growth phase were seeded at 3 × 10^4^ cells per well in 4‐well chamber slide (SPL) and incubated overnight at 37°C (Haaf et al. 1995). Cells were pretreated with Madecassoside for 1 h and then incubated with cisplatin (10 μM) for 2 h. The medium was replaced to remove cisplatin, and cells were further incubated for 12 h before fixation with methanol for 10 min. The fixed cells were permeabilized with PBS containing 0.1% Triton X‐100 for 5 min and blocked with PBS containing 5% BSA for 1 h at room temperature. Cells were then incubated overnight at 4°C with primary antibodies against RAD51 (1:300, Abcam) and γH2AX (1:500, Millipore) in PBS with 5% BSA, followed by incubation with Alexa Fluor secondary antibodies (1:1000, Abcam) for 1 h at room temperature. Nuclei were counterstained with 500 ng/mL 4,6‐diamidino‐2‐phenylindole (DAPI) for 5 min. After washing with PBS, coverslips were mounted on glass slides with an anti‐fade fluorescence mounting medium (Abcam). Images were obtained using a Nikon Eclipse TE2000‐U inverted microscope with a 40× objective and analyzed using the ImageJ software.

### 2.9 Statistical Analysis

All experiments were performed with at least three independent replicates. Data are expressed as mean ± standard deviation (SD). Statistical analyses were performed using GraphPad Prism software, version 8.0. For comparisons among multiple groups, a one‐way ANOVA followed by Tukey's post hoc test was applied. Statistical significance was set at *p* < 0.05.

## 3 Results and Discussion

### 3.1 Construction of the RAD51 Bioactivity Dataset

The bioactivity data for RAD51 were collected from ChEMBL, PubChem, and BindingDB. The dataset consisted of compounds targeting the DNA repair protein RAD51 homolog 1, identified by CHEMBL2034807 of ChEMBL, UniProt ID Q06609 of PubChem, and BindingDB. The SMILES string was standardized by sequentially performing structural sanitation, aromaticity perception, parent fragment selection, charge neutralization, and tautomer canonicalization processes using RDKit (Dec, 2024). To establish a robust binary classification and reduce label uncertainty of the training set, compounds were categorized using IC_50_ cutoffs. The initial library generated a final dataset comprising 626 unique compounds. Molecules with an IC_50_ of 2000 nM or less were classified as active molecules, and those with an IC_50_ of 30,000 nM or more were classified as inactive molecules. 39 active compounds with IC_50_ values ≤ 2000 nM and 441 inactive compounds (IC_50_ ≥ 30,000 nM) were identified, resulting in a highly imbalanced distribution. To reduce class imbalance and minimize potential bias toward the inactive class, 80 compounds were randomly selected from the 441 inactive compounds. The final dataset consisted of 39 active and 80 inactive compounds, with a total of 119 samples.

### 3.2 Performance Evaluation of Machine Learning Models

Five machine learning models (RF, XGBoost, SVM, KNN, and GLM) were systematically evaluated across four cumulative preprocessing pipelines: Basic, Basic + SMOTE, Basic + SMOTE + near‐zero variance (NZV) filtering, and Basic + SMOTE + NZV + correlation filtering (Corr). The ensemble‐based models, RF and XGBoost, showed better performance than the other algorithms and maintained stable predictive performance despite the diverse chemical features and small number of active compounds in the RAD51 dataset (Figure 2). The optimized RF model (SMOTE+NZV) showed an AUC value ranging from to 0.980–1.000. XGBoost showed slight differences depending on the preprocessing strategy, but overall showed good predictive performance, with accuracy ranging from 0.900 to 0.933 and an AUC of 0.920 to 0.990. In contrast, the support vector machine (SVM) and *k*‐nearest neighbor (KNN) models showed variable performance depending on the preprocessing conditions and had only moderate predictive ability. The performance of the SVM model improved after NZV and correlation‐based filtering, suggesting that removing low‐information and highly correlated variables helped improve its predictive ability. In contrast, the KNN model maintained a strong performance in Basic, NZV, and SMOTE preprocessing, but its predictive ability was noticeably reduced after correlation filtering. Among all the models evaluated, the generalized linear model (GLM) showed the weakest discriminant ability, with relatively low accuracy and AUC values close to 0.5, under the four preprocessing conditions. Overall, the RF model showed the most reliable performance under the cumulative Basic + SMOTE + NZV preprocessing pipeline, providing high accuracy, strong ROC‐AUC performance, and a high Cohen's kappa value (*κ* = 0.842). Based on this performance, the optimized RF model was selected as the final classifier and applied to virtual screening of the natural product library.

**FIGURE 2:**
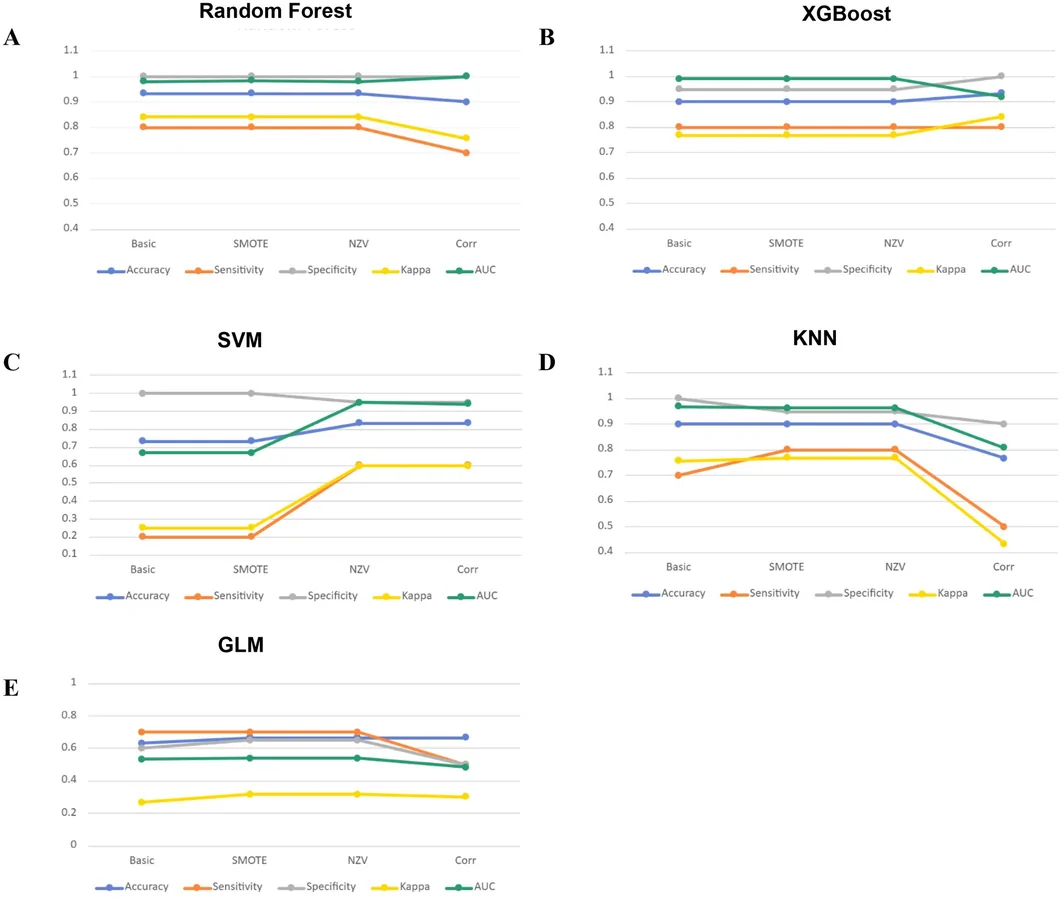
Performance comparison across preprocessing pipelines of five machine learning models for prediction of inhibition activity of RAD51. The line charts show the comparison of key performance metrics (Accuracy, Sensitivity, Specificity, Cohen's Kappa (*κ*), and AUC) between (A) Random Forest (RF), (B) XGBoost (XGB), (C) Support Vector Machine (SVM), (D) *k*‐Nearest Neighbors (KNN), and (E) Generalized Linear Model (GLM). The RF model integrated with SMOTE and NZV achieved high scores on almost all metrics and shows the most balanced and excellent performance. AUC, area under the receiver operating characteristic (ROC) curve; Corr, correlation‐based feature elimination; GLM, generalized linear model; IC_50_, Half‐maximal inhibitory concentration.; KNN, k‐nearest neighbors; NZV, near‐zero variance; RF, random forest; SMOTE, synthetic minority over‐sampling technique; SVM, support vector machine; XGBoost (XGB), extreme gradient boosting; *κ* (Kappa), Cohen's kappa coefficient.

### 3.3 Selection of the Optimal Classifier Based on Performance Stability

Among the four preprocessing strategies (Basic, SMOTE, NZV, and Corr) tested in this study, the RF model exhibited superior predictive performance, minimizing variations in sensitivity and specificity across all pipelines (Table S1 and Figure 2). XGBoost (XGB) showed competitive results under Corr conditions compared to the RF model, but with relatively large fluctuations in Cohen's Kappa and specificity. The radar chart compares the best and worst performance of each algorithm and shows that the RF model maintained the most stable performance across different preprocessing conditions (Figure 3). The SVM and GLM exhibited a sharp decline in performance under certain conditions. The SVM showed a distinct contraction to the center of the chart, with the sensitivity remaining at the level of 0.2 and the kappa index (kappa) at the level of 0.25 in the default and SMOTE settings. In the case of the KNN model, the sensitivity showed some improvement from 0.7 to 0.8 when applying class rebalancing (SMOTE). However, in the correlation filtering (Corr) pipeline, all performance indicators underwent sharp degradation. This indicates that the SVMs and KNN models were more susceptible to preprocessing‐dependent changes, and the predictions may be inconsistent depending on certain conditions during the preprocessing.

**FIGURE 3:**
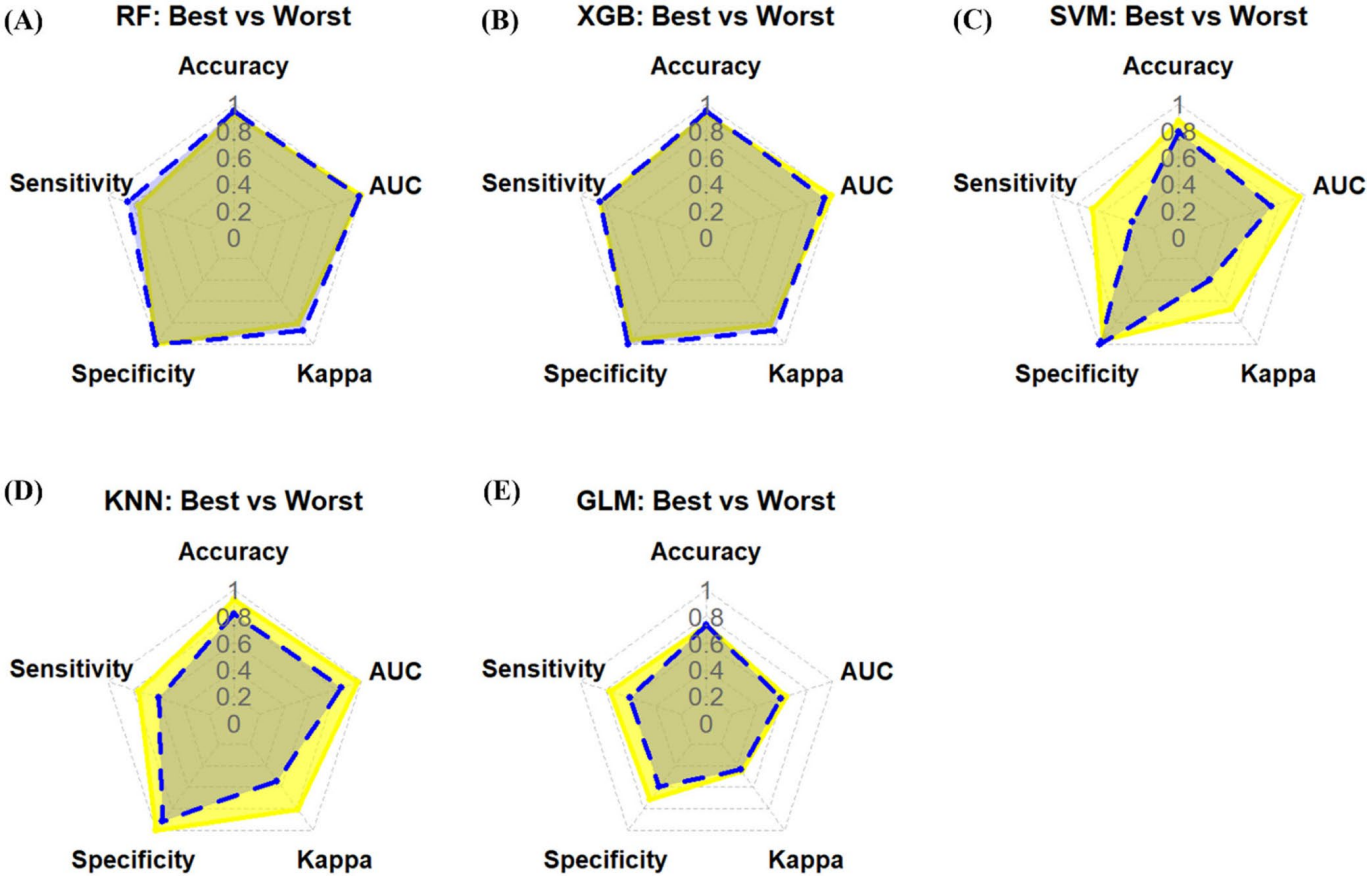
Comparison of the performance stability of each pre‐processing step for five machine learning models. The radar chart visualizes the profile of the ‘Best (Yellow Area)’ and ‘Worst (Blue Dotted Line)’ performance of each algorithm during the four pre‐processing pipelines (Default, SMOTE, NZV, Corr). The performance evaluation was based on five metrics: Accuracy, AUC, Cohen's Kappa (*κ*), specificity, and sensitivity. (A, B) Ensemble models (RF and XGB): Both models showed almost identical patterns of optimal performance and lowest performance profiles. These suggest that the model maintained extremely stable and robust predictive power regardless of changes in preprocessing strategies. (C) SVM: At the lowest performance setting (default and SMOTE conditions), the sensitivity (0.2) and kappa index (0.25) were rapidly reduced and the polygon was greatly contracted to the center. This proves that the performance deviation of the SVM model was very large depending on whether or not an appropriate features election was made. (D) There was a positive effect of improving sensitivity when applying KNN and SMOTE, but in the correlation filtering (Corr) pipeline, all indicators collapsed and showed an unstable prediction trend. (E) GLM: This model recorded the narrowest profile and the lowest discriminant power, clearly showing the limitations of linear models in learning complex nonlinear relationships in this dataset.

### 3.4 Virtual Screening and Hit Identification

An optimized RF (NZV+ SMOTE) model was used to screen a library of 1142 natural products. With the predicted probability value (pred_prob) set to 0.5 as a threshold, 188 compounds were identified as candidates, of which 10 commercially available compounds were selected for the final docking simulations among the top 15 compounds with high probability values. Docking simulations were performed targeting the ANP‐binding pocket of RAD51 (PDB ID: 5NP7), where the co‐crystallized ligand ANP was located. Geranin had the lowest binding energy of −10.4 kcal/mol at the binding site (Table 2). However, among the nine predictive poses generated by AutoDock, Geraniin showed rapidly increasing energy variability in certain binding poses. Madecassoside showed a low binding energy at −8.9 kcal/mol and confirmed that all nine poses produced were evenly distributed within a narrow energy deviation. In addition to the superiority of the simple binding energy, analysis of intermolecular interactions through docking poses confirmed that Madecassoside showed a stable binding pattern within the ANP‐binding pocket (PDB ID: 5NP7) of RAD51 (Figure 4). The triterpenoid skeleton forms a structural basis by deeply settling in the hydrophobic cavity formed by Gln135, Thr134. The glycoside chain of the molecule forms a dense hydrogen bonding network with residues such as Ser223, Asn267, and Lys284 to maximize binding affinity. As a result, Madecassoside was selected as the final hit compound to confirm its inhibitory activity on RAD51 foci formation using cell‐based assays.

**TABLE 2:** Prediction probability and molecular docking energy of the top 10 natural product candidates selected through virtual screening. This table lists 10 compounds that can be purchased among the top candidates whose high probability score is predicted by the optimized RF (NZV+ SMOTE) model. For each candidate compound, probability score and molecular docking score (kcal/mol) are summarized. The lower the docking energy, the higher the binding affinity with the target protein, and in this study, Madecassoside was selected as the final active material in consideration of the prediction probability and structural stability comprehensively.

Rank	Compound name	Probability score	Docking score (kcal/mol)
1	Mogrol	0.640	−7.6
2	Punicalin	0.624	−8.6
3	Bryodulcosigenin	0.624	−7.4
4	Geraniin	0.610	−9.4
5	Corilagin	0.606	−8.0
6	Madecassoside	0.604	−8.9
7	Plantamajoside	0.600	−7.3
8	Protodioscin	0.598	−8.6
9	(20*R*)‐Protopanaxatriol	0.596	−6.6
10	(20*S*)‐Protopanaxatriol	0.596	−6.7

**FIGURE 4:**
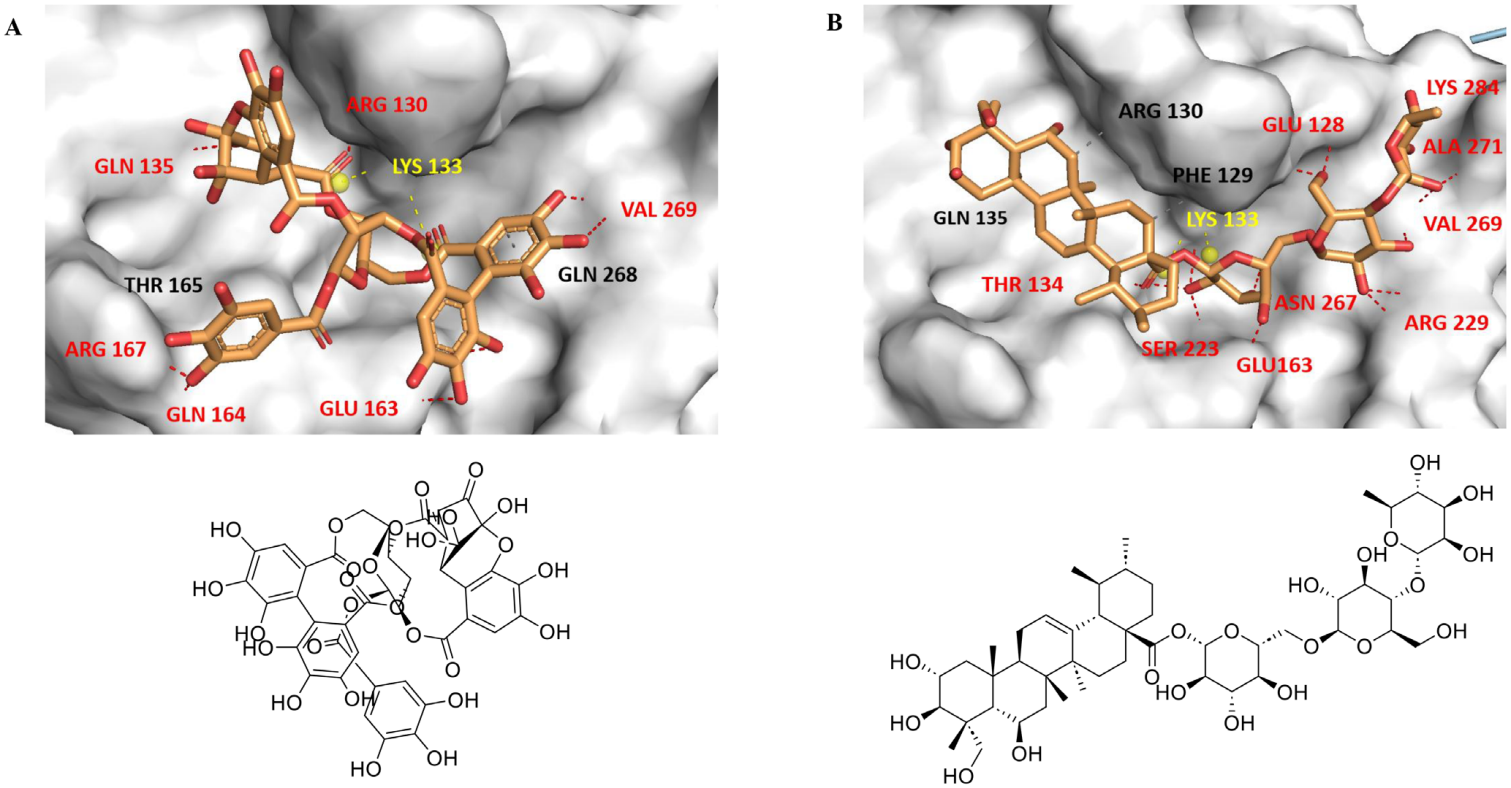
Molecular docking interactions of Geraniin (A) and Madecassoside (B) within the ATP‐binding site of RAD51 (PDB ID: 5NP7). This figure shows the binding mode of virtual screening candidates performed on the ATP binding site (ANP pocket) of PDB ID 5NP7 structure. Geraniin exhibited the highest docking score and formed multiple hydrogen‐bond interactions with residues including Gln135, Arg130, and Lys133. Madecassoside, selected for further biological evaluation, formed interactions with residues such as Gln135, Thr134, Ser223, Asn267, and Lys284, suggesting favorable binding interactions within the ATP‐binding pocket of RAD51.

### 3.5 Madecassoside Inhibits RAD51 Foci Formation Induced by Cisplatin in Cancer Cells

We evaluated whether Madecassoside enhanced the sensitivity of MDA‐MB‐231 breast cancer cells to clinically approved anticancer agents, including cisplatin. Clonogenic survival assays demonstrated that treatment with Madecassoside (5 μM) increased the sensitivity of MDA‐MB‐231 cells to cisplatin (Figure 5A). Notably, treatment with Madecassoside alone did not significantly affect cell viability (Figure 5B). RAD51 forms nuclear foci in response to DNA damage induced by chemical agents or radiation (Salentin et al. 2015). Formation of RAD51 foci is a critical step in homologous recombination (HR)‐mediated DNA repair, and failure to repair DNA damage results in reduced cellular survival. To determine whether the antiproliferative effect of Madecassoside is mediated through inhibition of RAD51 foci formation, we examined the effects of Madecassoside on RAD51 foci formation and the DNA damage marker γH2AX following treatment with a DNA‐damaging agent. MDA‐MB‐231 cells were treated with either DMSO or Madecassoside for 1 h before cisplatin exposure. As expected, cisplatin treatment increased the number of RAD51 foci per nucleus, whereas Madecassoside treatment suppressed cisplatin‐induced RAD51 foci formation (Figure 5C,D). These results indicated that Madecassoside exerts a biological effect on MDA‐MB‐231 breast cancer cells by inhibiting DNA damage‐induced RAD51 foci formation.

**FIGURE 5:**
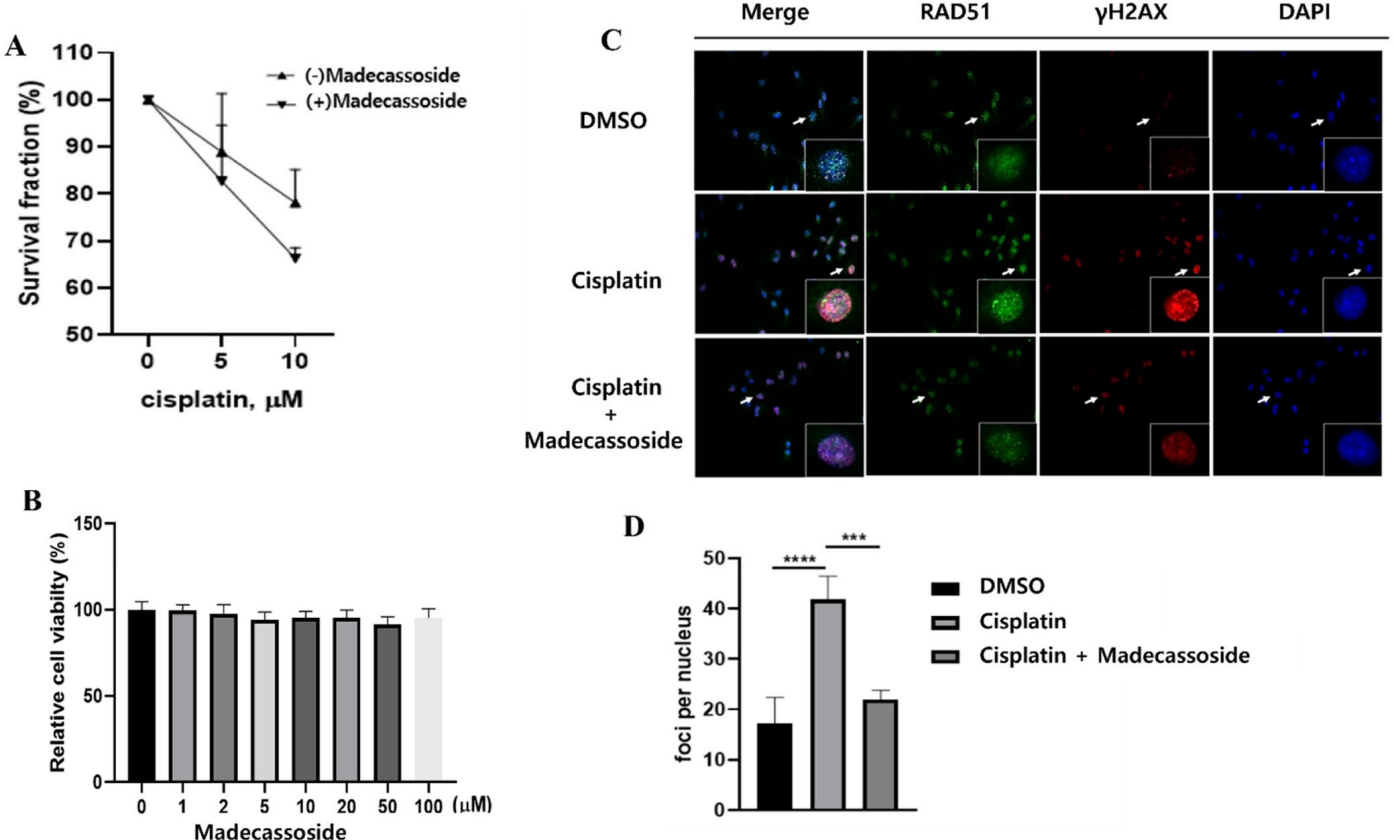
Madecassoside inhibits RAD51 foci formation in MDA‐MB‐231 cells. (A) Clonogenic survival of MDA‐MB‐231 cells treated with cisplatin in the absence (▲) or presence (▼) of Madecassoside (5 μM). (B) Cytotoxicity of Madecassoside in MDA‐MB‐231 cells. All experiments were performed at least three times independently. Error bars represent the standard deviation (SD). (C, D) Image analysis of DNA damage and repair markers following Madecassoside treatment. MDA‐MB‐231 cells were treated with DMSO (upper and middle panels) or Madecassoside (10 μM; lower panel) for 1 h, then cells were exposed to cisplatin (10 μM) for 2 h (middle and lower panels) and stained 12 h later. RAD51 foci and γH2AX signals were visualized by immunostaining using anti‐RAD51 and anti‐γH2AX antibodies. Nuclei were counterstained with DAPI. Quantification shows fraction of cells with ≥ 10 RAD51 foci. Data are presented as mean ± SD from at least three independent biological experiments. ***p < 0.001; ****p < 0.0001.

## 4 Discussion

Homologous recombination is an essential DNA repair pathway responsible for maintaining genomic stability through accurate repair of DNA double‐strand breaks. RAD51 is a key protein involved in DNA replication, repair, and recombination processes that mediate HR during DNA double‐stranded cleavage repair to promote strand invasion and strand exchange between homologous DNA strands. Abnormal RAD51 overexpression has been reported in several cancers and is associated with improved DNA repair capacity, treatment resistance, and decreased clinical outcomes. Although RAD51 is attracting attention as a promising therapeutic target because of its association with DNA damage repair mechanisms that protect cancer cells from radiation and chemotherapy, the development of RAD51 inhibitors is still in its early stages.

In this study, we built a pipeline that incorporated machine learning and structure‐based discoveries to identify novel RAD51 inhibitors. We obtained biological activity datasets from the ChEMBL, PubChem, and BindingDB databases and systematically evaluated their predictive performance by applying four preprocessing strategies to each of five machine learning models. Among the five machine learning models, the RF model performed best under the cumulative Basic + SMOTE + NZV preprocessing pipeline, achieving high accuracy, excellent AUC values, and substantial agreement based on Cohen's kappa. These results suggest that the ensemble‐based algorithm can effectively predict the complex relationship between molecular descriptors and RAD51 inhibitory activity. The final selected optimized RF model (NZV + SMOTE) was applied to virtual screening of in‐house natural product libraries, and based on the results of the docking simulation, Madecassoside was determined to be a promising RAD51 candidate compound.

Docking simulations of the ATP‐binding pockets of RAD51 (PDB ID: 5NP7) revealed that Madecassoside forms hydrogen bonds with residues Gln135, Ser223, and Asn267. Furthermore, it inhibits ATP‐dependent RAD51 activation and filament stabilization in a manner that is different from that of known RAD51 inhibitors. Madecassoside was not cytotoxic when administered alone and improved the sensitivity of MDA‐MB‐231 breast cancer cells to cisplatin. Madecassoside also inhibited cisplatin‐induced RAD51 foci formation, indicating potential inhibition of RAD51‐mediated homologous recombination repair activity. As RAD51 foci formation is an important step in HR‐dependent DNA repair, these findings suggest that Madecassoside may impair the DNA damage response, allowing cancer cells to react sensitively to DNA damage factors. This study has several limitations. First, the size of the final established dataset was relatively small because of the limitations of the experimentally verified RAD51 inhibitor data. Although various preprocessing methods and cross‐validation strategies were used to improve model robustness, it is believed that larger datasets can further improve the predictive performance and generalization capabilities. Second, ATP‐binding‐based inhibition mechanisms have been proposed, mainly based on docking simulations and indirect biological results. Therefore, additional biochemical experiments, such as ATPase activity measurements or direct binding analyses, are required. Finally, this study did not include in vivo testing or pharmacokinetic characteristic analysis, which should be supplemented in future studies. Nevertheless, this study showed that an approach combining machine learning‐based virtual screening with experimental validation can be an effective strategy for discovering novel RAD51 inhibitors. Furthermore, this study demonstrated that an approach incorporating AI‐based screening, structure‐based analysis, and functional cell‐based experiments could accelerate early drug development. Thus, Madecassoside is a promising lead scaffold for the development of RAD51 inhibitors and DNA repair‐targeted anticancer agents.

## Author Contributions

**Minju Oh:** investigation, writing – original draft, methodology, visualization, validation, software, data curation. **Mi‐Young Jeong:** methodology, validation, writing – original draft, formal analysis. **Jiwon Choi:** conceptualization, investigation, funding acquisition, writing – original draft, methodology, validation, visualization, writing – review and editing, software, formal analysis, project administration, data curation, supervision, resources.

## Funding

This research was supported by grants from the National Research Foundation of Korea, funded by the Korean government (MSIT) (no. RS‐2023‐00280171).

## Conflicts of Interest

The authors declare no conflicts of interest.

## Supporting information


**Table S1:** Detailed classification metrics for five machine learning models across different preprocessing pipelines. The performance metrics for five machine learning classifiers were evaluated on the independent test set across four subsets.

## Data Availability

The data that support the findings of this study are available in ChEMBL at https://www.ebi.ac.uk/chembl/. These data were derived from the following resources available in the public domain: ChEMBL, https://www.ebi.ac.uk/chembl; PubChem, https://pubchem.ncbi.nlm.nih.gov/; BindingDB, https://www.bindingdb.org/rwd/bind/index.jsp.
