# Supplementary material for: Machine Learning‐Guided Identification of Madecassoside as a Potential RAD51 Modulator That Sensitizes Breast Cancer Cells to Cisplatin
Source: Chem Biol Drug Des. 2026 Oct 13;108(4):e70416. doi: 10.1111/cbdd.70416 (PMC13661784; doi:10.1111/cbdd.70416)
Supplement: Supplementary file 1 [file CBDD-108-e70416-s001.docx]

**Table S1.** Detailed classification metrics for five machine learning models across different preprocessing pipelines. The performance metrics for five machine learning classifiers were evaluated on the independent test set across four subsets.

| Model | Subset | Accuracy | Sensitivity | Specificity | Kappa (κ) | AUC |
| --- | --- | --- | --- | --- | --- | --- |
| RF | Basic | 0.933 | 0.800 | 1.000 | 0.842 | 0.980 |
|  | SMOTE | 0.933 | 0.800 | 1.000 | 0.842 | 0.985 |
|  | NZV | 0.933 | 0.800 | 1.000 | 0.842 | 0.980 |
|  | Corr | 0.900 | 0.700 | 1.000 | 0.757 | 1.000 |
| XGBoost | Basic | 0.900 | 0.800 | 0.950 | 0.769 | 0.990 |
|  | SMOTE | 0.900 | 0.800 | 0.950 | 0.769 | 0.990 |
|  | NZV | 0.900 | 0.800 | 0.950 | 0.769 | 0.990 |
|  | Corr | 0.933 | 0.800 | 1.000 | 0.842 | 0.920 |
| SVM | Basic | 0.733 | 0.200 | 1.000 | 0.250 | 0.667 |
|  | SMOTE | 0.733 | 0.200 | 1.000 | 0.250 | 0.667 |
|  | NZV | 0.833 | 0.600 | 0.950 | 0.595 | 0.950 |
|  | Corr | 0.833 | 0.600 | 0.950 | 0.595 | 0.940 |
| KNN | Basic | 0.900 | 0.700 | 1.000 | 0.757 | 0.974 |
|  | SMOTE | 0.900 | 0.800 | 0.950 | 0.769 | 0.963 |
|  | NZV | 0.900 | 0.800 | 0.950 | 0.769 | 0.963 |
|  | Corr | 0.767 | 0.500 | 0.900 | 0.432 | 0.801 |
| GLM | Basic | 0.633 | 0.700 | 0.600 | 0.267 | 0.533 |
|  | SMOTE | 0.667 | 0.700 | 0.650 | 0.318 | 0.540 |
|  | NZV | 0.667 | 0.700 | 0.650 | 0.318 | 0.540 |
|  | Corr | 0.667 | 0.500 | 0.500 | 0.302 | 0.483 |

Abbreviations: RF: Random Forest; XGBoost: Extreme Gradient Boosting; SVM: Support Vector Machine; KNN: *k*-Nearest Neighbor; GLM: Generalized Linear Model. Subsets: Basic (Basic preprocessing, including removal of missing and infinite values); SMOTE (Synthetic Minority Over-sampling Technique); NZV (Near-Zero Variance filtering); Corr (Correlation-based feature elimination, R>0.95). Metrics: Accuracy (ACC); Sensitivity (Recall); Specificity; Cohen’s Kappa (κ); Area Under the Receiver Operating Characteristic Curve (AUC).
